# Draft Genome Sequences of Three *Pectobacterium* Strains Causing Blackleg of Potato: *P. carotovorum* subsp. *brasiliensis* ICMP 19477, *P. atrosepticum* ICMP 1526, and *P. carotovorum* subsp. *carotovorum* UGC32

**DOI:** 10.1128/genomeA.00874-15

**Published:** 2015-08-06

**Authors:** Preetinanda Panda, Mark W. E. J. Fiers, Ashley Lu, Karen F. Armstrong, Andrew R. Pitman

**Affiliations:** a Bio-Protection Research Centre, Lincoln University, Canterbury, New Zealand; b The New Zealand Institute for Plant & Food Research Limited, Lincoln, Canterbury, New Zealand

## Abstract

Blackleg is a disease caused by several species of *Pectobacterium* that results in losses to potato crops worldwide. Here, we report the draft genomes of three taxonomically and geographically distinct blackleg-causing strains of *Pectobacterium*: *P. carotovorum* subsp. *brasiliensis* ICMP 19477, *P. atrosepticum* ICMP 1526, and *P. carotovorum* subsp. *carotovorum* UGC32. Comparison of these genomes will support the identification of common traits associated with their capacity to cause blackleg.

## GENOME ANNOUNCEMENT

Historically, *Pectobacterium atrosepticum* was the pathogen considered responsible for blackleg, an economically important disease of potato (1). Surveys to identify the source of blackleg in the field, however, led to the isolation of numerous species and subspecies of *Pectobacterium* from disease lesions including *P. carotovorum* subsp. *brasiliensis* (2) and *P. carotovorum* subsp. *carotovorum* (3). The isolation of divergent *Pectobacterium* species causing blackleg suggested that these bacteria probably harbor common virulence determinants that enable them to cause stem infection of their host.

In this study, the draft genome sequences were obtained for three taxonomically and geographically distinct strains of *Pectobacterium* to decode genetic features associated with their ability to cause blackleg. FLX 454-platform pyrosequencing (4) was conducted to attain the genomes of an aggressive blackleg-causing *P. carotovorum* subsp. *brasiliensis* strain from New Zealand (ICMP 19477) (5) and the type strain for *P. atrosepticum*, ICMP 1526 (ATCC 33260), from the United Kingdom. Illumina paired-end sequencing technology (Illumina, Inc., CA, USA) was used to ascertain the genome sequence of UGC32, an unusual blackleg-causing strain from Peru identified as *P. carotovorum* subsp. *carotovorum* (6).

The genome sequencing of ICMP 19477 and ICMP 1526 was performed using an FLX 454 sequencer by the Liverpool Advanced Genomics Facility (Liverpool University, United Kingdom), while the genome of UGC32 was sequenced using a HiSeq 2000 system to generate Illumina paired-end reads (Axeq Technologies, USA). The 454 sequence reads were trimmed and assembled into large contigs using the NewblerGS *de novo* assembler (454 Life Science) and contigs <250 bp and with low coverage were removed from the assembly using Tablet (7). Gap-filling PCR followed by Sanger sequencing of PCR amplicons was carried out to close gaps between unlinked contigs for ICMP 19477 and ICMP 1526. Reassembly of the 454 and Sanger sequences using NewblerGS resulted in genome coverages of ~50× and ~40× for ICMP 19477 and ICMP 1526, respectively. The removal of adapter sequences and trimming of the Illumina sequence reads for UGC32 using FastQC (Baraham Bioinformatics, United Kingdom), followed by assembly of the trimmed reads using SOAP *de novo* v2.04 (8), generated a draft genome sequence with a coverage of ~600×.

Genome annotation for ICMP 19477, ICMP 1526, and UGC32 was performed using the PGAAP annotation pipeline (http://www.ncbi.nlm.nih.gov/genome/annotation_prok/). The estimated draft genome sizes for these bacteria were as follows: ICMP 19477 is 4,979,068 bp comprising 35 contigs with 52.1% G+C content; ICMP 1526 is 4,873,856 bp comprising 38 contigs with 50.8% G+C content; and UGC32 is 4,797,741 bp comprising 28 contigs with 51.1% G+C content. Annotations predicted 4,435, 4,409, and 4,082 coding sequences and 67, 68, and 74 tRNAs for ICMP 19477, ICMP 1526, and UGC32, respectively. Various loci associated with virulence were present in all three genomes, suggesting that a more detailed comparison of these genomes will enable the identification of common genomic regions associated with blackleg in *Pectobacterium*.

### Nucleotide sequence accession numbers.

The draft genome sequences of ICMP 19477, ICMP 1526, and UGC32 have been deposited in the GenBank database under the accession numbers ALIU00000000, ALIV00000000, and AODU00000000, respectively. The versions described in this paper are the first versions, ALIU01000000, ALIV01000000, and AODU01000000.
